# Single centre experience of the application of self navigated 3D whole heart cardiovascular magnetic resonance for the assessment of cardiac anatomy in congenital heart disease

**DOI:** 10.1186/s12968-015-0156-7

**Published:** 2015-07-09

**Authors:** Pierre Monney, Davide Piccini, Tobias Rutz, Gabriella Vincenti, Simone Coppo, Simon C. Koestner, Nicole Sekarski, Stefano Di Bernardo, Judith Bouchardy, Matthias Stuber, Juerg Schwitter

**Affiliations:** Division of Cardiology and Cardiac MR Center, University Hospital of Lausanne (CHUV), Lausanne, Switzerland; Advanced Clinical Imaging Technology, Siemens Healthcare, Lausanne, Switzerland; Department of Radiology, University Hospital and University of Lausanne, Lausanne, Switzerland; Center for Biomedical Imaging and Center for Cardiovascular Magnetic Resonance Research, University of Lausanne, Lausanne, Switzerland; Pediatric Cardiology Unit, University Hospital of Lausanne (CHUV), Lausanne, Switzerland

**Keywords:** Congenital heart disease, Cardiovascular magnetic resonance, Self-navigation, Navigator, Free-breathing

## Abstract

**Background:**

For free-breathing cardiovascular magnetic resonance (CMR), the self-navigation technique recently emerged, which is expected to deliver high-quality data with a high success rate. The purpose of this study was to test the hypothesis that self-navigated 3D-CMR enables the reliable assessment of cardiovascular anatomy in patients with congenital heart disease (CHD) and to define factors that affect image quality.

**Methods:**

CHD patients ≥2 years-old and referred for CMR for initial assessment or for a follow-up study were included to undergo a free-breathing self-navigated 3D CMR at 1.5T. Performance criteria were: correct description of cardiac segmental anatomy, overall image quality, coronary artery visibility, and reproducibility of great vessels diameter measurements. Factors associated with insufficient image quality were identified using multivariate logistic regression.

**Results:**

Self-navigated CMR was performed in 105 patients (55 % male, 23 ± 12y). Correct segmental description was achieved in 93 % and 96 % for observer 1 and 2, respectively. Diagnostic quality was obtained in 90 % of examinations, and it increased to 94 % if contrast-enhanced. Left anterior descending, circumflex, and right coronary arteries were visualized in 93 %, 87 % and 98 %, respectively. Younger age, higher heart rate, lower ejection fraction, and lack of contrast medium were independently associated with reduced image quality. However, a similar rate of diagnostic image quality was obtained in children and adults.

**Conclusion:**

In patients with CHD, self-navigated free-breathing CMR provides high-resolution 3D visualization of the heart and great vessels with excellent robustness.

## Background

Congenital heart disease (CHD) is frequent with an estimated prevalence of 0.4 % in adults [1]. Considerable progress in the treatment has been achieved with 84 % of infants being expected to reach adulthood [2]. In addition to echocardiography, cardiovascular magnetic resonance (CMR) plays an important role in the initial assessment and in the follow-up [3, 4] and recommendations have been published to guide CMR examinations in these patients [5–8].

For the assessment of intra-thoracic anatomy, free-breathing approaches are increasingly performed, yielding a 3D representation of the heart, which can be reformatted off-line in any slice orientation. For such acquisitions, respiratory gating is needed, which is traditionally performed by using a beam-navigator placed on the dome of the right hemi-diaphragm [9]. This approach assumes a fixed coupling ratio between diaphragmatic and cardiac displacement during respiration. As an alternative, a motion correction algorithm based on cardiac self-navigation has been developed [10], where the position of the heart during the respiratory cycle is extracted directly from the data. In particular, k-space lines oriented in a superior-inferior direction allow to identify the position of the ventricular blood pool signal, and thus for a direct 1D correction of the heart position during free breathing, without relying on the assumption of a constant diaphragmatic-heart displacement correlation. Furthermore, a 3D radial acquisition scheme ensures an intrinsic robustness to motion and to undersampling and fold-over artifacts. Up until now, studies in small numbers of volunteers [11, 12] and patients [13] have been reported using this technique for coronary imaging. The aims of this work were to test the hypothesis that self-navigated 3D CMR with high isotropic spatial resolution enables the reliable assessment of cardiovascular anatomy in CHD patients and to define factors that affect image quality.

## Methods

### Patient selection

Between June 2012 and September 2013, all patients ≥2 years old with congenital disease involving the heart or the great vessels and referred for CMR were considered for inclusion. Patients with irregular heart rhythms were excluded (n = 1). Children aged <8 years were usually scanned under general anesthesia with controlled breathing. The study protocol was approved by the “Commission Cantonale d’Ethique de la Recherche sur l’Etre Humain” (protocol 70/14) and patients or their parents gave informed consent.

### Standard CMR imaging

Patients were scanned at 1.5T (MAGNETOM Aera, Siemens AG, Healthcare Sector, Erlangen, Germany) with a 30-channel phased-array coil. The imaging protocol was selected according to the specific malformation [5–8]. Contrast medium (CM) was used in 97 (87 %) patients (Gadobutrol, Gadovist®, Bayer AG, Zurich, Switzerland, dose: 0.2mmol/kg). No heart rate (HR) lowering drugs were given.

### Free-breathing 3D self-navigated CMR acquisition

Data acquisition was performed with a 3D radial SSFP prototype sequence with a specific readout arrangement following a spiral phyllotaxis pattern [14] adapted for self-navigation [12]. All measurements were segmented and ECG-triggered and a T_2_-preparation pulse preceded the fat-saturated read-out. With this self navigation strategy, the first k-space line acquired at each segment is consistently oriented along a superior-inferior direction. The 1D Fourier transform of such k-space lines allows for the detection of the position of the ventricular blood pool at each heartbeat [12]. This information represents the respiratory motion of the heart over the whole data acquisition and is used to “shift” all k-space data in the superior-inferior direction allowing for 100 % scan efficiency. If intravenous contrast medium was injected, self-navigated acquisition started after injection. The imaging parameters were as follows: TR/TE 3.1/1.56ms, field-of-view 182–220mm, matrix 192, receiver bandwidth 898Hz/pixel, isotropic spatial resolution (both acquired and reconstructed) 0.88–1.15mm, 12′417–15′050 radial readouts over 377–953 heartbeats (Table 1). The trigger delay was visually identified as the most quiescent mid-diastolic period on a four-chamber cine loop, but an end-systolic acquisition window could also be chosen, especially for higher HR. The temporal resolution of the sequence was adapted to the HR at the discretion of the operator, but reduction of the acquisition window duration always led to an increase in shots (number of heart beats of image acquisition) to acquire a minimum of 12′000 k-space profiles.

**Table 1:** Demographics and imaging parameters of the 3D-self-navigated acquisition: grouping according image quality categories

		Total cohort	Quality grade 1	Quality grade 2	Quality grade 3	Quality grade 4	Quality grade 5	*P*
		N = 111	N = 1	N = 10	N = 22	N = 41	N = 37	
**Baseline characteristics**								
Age	years	23.4 ± 12.2	8.6	22.7 ± 14.2	23.6 ± 15.7	25.6 ± 12.7	21.4 ± 0.1	0.43
Age <16 y	N (%)	30 (27.0 %)	1 (100 %)	4 (40 %)	9 (40.9 %)	9 (22.0 %)	7 (18.9 %)	0.10
Male gender	N (%)	61 (55.0 %)	0 (0 %)	4 (40 %)	13 (59.1 %)	22 (53.7 %)	22 (59.5 %)	0.66
HR	bpm	75.4 ± 14.3	88	87.9 ± 16.3	76.3 ± 15.8	73.2 ± 12.0	73.5 ± 13.9	**<0.05**
SDRR^a^	ms	90 ± 87	48	91 ± 109	99 ± 87	108 ± 93	66 ± 72	0.31
Height	cm	160.6 ± 23.6	147	148.8 ± 33.2	152.3 ± 31.8	166.2 ± 14.4	163.0 ± 21.9	0.08
Weight	kg	58.0 ± 21.1	43	47.5 ± 24.6	53.6 ± 27.5	62.3 ± 16.8	59.1 ± 19.6	0.22
Body Mass Index	kg/m^2^	21.5 ± 4.9	19.9	19.44 ± 6.3	21.1 ± 6.0	22.2 ± 4.5	21.5 ± 4.1	0.59
EDVi^b^ of the systemic ventricle	ml/m^2^	82.4 ± 25.0	63	98.7 ± 45.9	76.1 ± 14.6	80.0 ± 18.9	84.8 ± 27.0	0.14
EF of the systemic ventricle	%	58.2 ± 8.5	60	49.3 ± 12.2	58.6 ± 10.0	59.5 ± 6.7	58.9 ± 7.0	**<0.05**
Baseline characteristics								
Complex malformation	N (%)	49 (44.1 %)	0 (0 %)	8 (80.0 %)	11 (50.0 %)	13 (31.7 %)	17 (46.0 %)	**<0.05**
- Tetralogy of Fallot		20 (18.0 %)	0	3	3	8	6	
- d-TGA^c^		13 (11.7 %)	0	2	3	3	5	
- Fontan circulation		3 (2.7 %)	0	0	2	0	1	
-Other complex		13 (11.7 %)	0	3	3	2	5	
Non-complex malformation	N (%)	62 (55.9 %)	1 (100 %)	2 (20 %)	11 (50 %)	28 (68.3 %)	20 (54.0 %)	**<0.05**
- Aortic dilatation		22 (19.8 %)	0	1	3	10	8	
- Coarctation aorta		7 (6.3 %)	0	0	0	5	2	
- After Ross operation		8 (7.2 %)	0	0	2	4	2	
- Septal defect		5 (4.5 %)	1	1	1	2	0	
- Abnormal venous return		8 (7.2 %)	0	0	1	3	4	
- Other non-complex		12 (10.8 %)	0	0	4	4	4	
Corrected malformation	N (%)	76 (68.5 %)	0 (0 %)	5 (50.0 %)	14 (63.6 %)	28 (68.3 %)	29 (78.4 %)	0.21
Characteristics of the 3D sequence								
Field of view	mm	205 ± 14	199	206 ± 13	204 ± 21	205 ± 11	206 ± 11	0.95
Isotropic resolution	mm	1.01 ± 0.09	0.96	1.0 ± 0.09	1.02 ± 0.10	1.01 ± 0.09	1.01 ± 0.09	0.9
Acquisition window	ms	68.2 ± 21.2	45	54 ± 25.4	67.8 ± 26.3	72.5 ± 17.5	68.3 ± 19.4	0.11
Systolic acquisition	N (%)	20 (18.0 %)	1 (100)	2 (20.0)	2 (9.1)	4 (9.8)	11 (29.7)	**<0.05**
Number of heart beats	N	705.3 ± 299.0	898	975 ± 495	715 ± 372	637 ± 201	698 ± 245	**<0.05**
Number of segments	N	22.7 ± 7.1	15	18.0 ± 8.5	22.6 ± 8.8	24.2 ± 5.8	22.8 ± 6.5	0.11
Number of scan lines	N × 1000	14.2 ± 2.1	13.4	14.3 ± 1.9	13.5 ± 2.7	14.4 ± 1.9	14.5 ± 1.9	0.48
Scan duration	min	9.5 ± 3.1	10.2	11.2 ± 3.7	9.3 ± 3.1	9.0 ± 2.4	9.6 ± 3.5	0.38
Use of contrast	N (%)	97 (87.4 %)	0 (0 %)	6 (60.0 %)	21 (95.5 %)	33 (80.5 %)	37 (100 %)	**<0.001**

^a^SDRR = standard deviation of the RR interval during image acquisition

^b^EDVi = end-diastolic volume index

^c^d-TGA = d-transposition of the great arteries

### Assessment of image quality

Four components were assessed in each dataset: 1) Accuracy and reproducibility of the segmental morphological analysis, 2) Grading of image quality, 3) Coronary artery visualization, and 4) Reproducibility of great vessels diameter measurements.

### Segmental morphology and residual structural defects

The reading of the 3D images was performed in two steps. In a first step, the aim was to assess whether image quality was high enough to allow for a reproducible description of the segmental cardiac anatomy without any a priori knowledge of the diagnosis and the previous surgical corrections. Two experienced readers, blinded to diagnosis and previous surgical history, independently performed a systematic segmental description of the cardiac anatomy from the 3D-self-navigated dataset only (for elements analyzed, see Table 2). This analysis was performed at least 6 months after the clinical report had been written.

**Table 2:** Accuracy and reproducibility of segmental cardiac analysis with 3D-self-navigated CMR

N = 105 patients	N with abnormal findings	Agreement Obs 1 vs Gold standard	Agreement Obs 2 vs Gold standard	Agreement Obs 1 vs Obs 2
1. Atrial situs	1	100 %	100 %	100 %
2. Position of the apex	1	100 %	100 %	100 %
3. Atrial segment morphology	11	100 %	100 %	100 %
4. Atrio-ventricular connection	2	99 %	99 %	100 %
5. Ventricular segment morphology	6	100 %	99 %	99 %
6. Ventriculo-arterial connection	18	99 %	98.1 %	99 %
7. Anatomy of the thoracic aorta	25	100 %	100 %	100 %
8. Anatomy of the venous return	16	100 %	100 %	100 %
9. Intracardiac shunts	12	98.1 %	99 %	97.1 %
10. Course of the coronary arteries	4	100 %	98.1 %	98.1 %
**Complete segmental anatomy**				
(Agreement = all 10 elements correctly evaluated)		**96.2 %**	**93.3 %**	**93.3 %**

Obs: Observer

In the second step, the aim was the assessment of the ability of the 3D sequence to detect residual uncorrected structural defects. A second reading was performed, which corresponded to the routine clinical situation, with the readers un-blinded to the diagnosis and the surgical history of the patients. In order to establish the reference diagnosis in each patient, the following sources were used: surgical and/or neonatal catheterization reports (available for 88 % of the operated patients), previous cardiac catheterization, echocardiography and previous CMR reports. The results of each individual reader were then compared with the reference diagnosis and inter-reader agreement was determined.

### CMR image quality grading

**Fig. 1:**
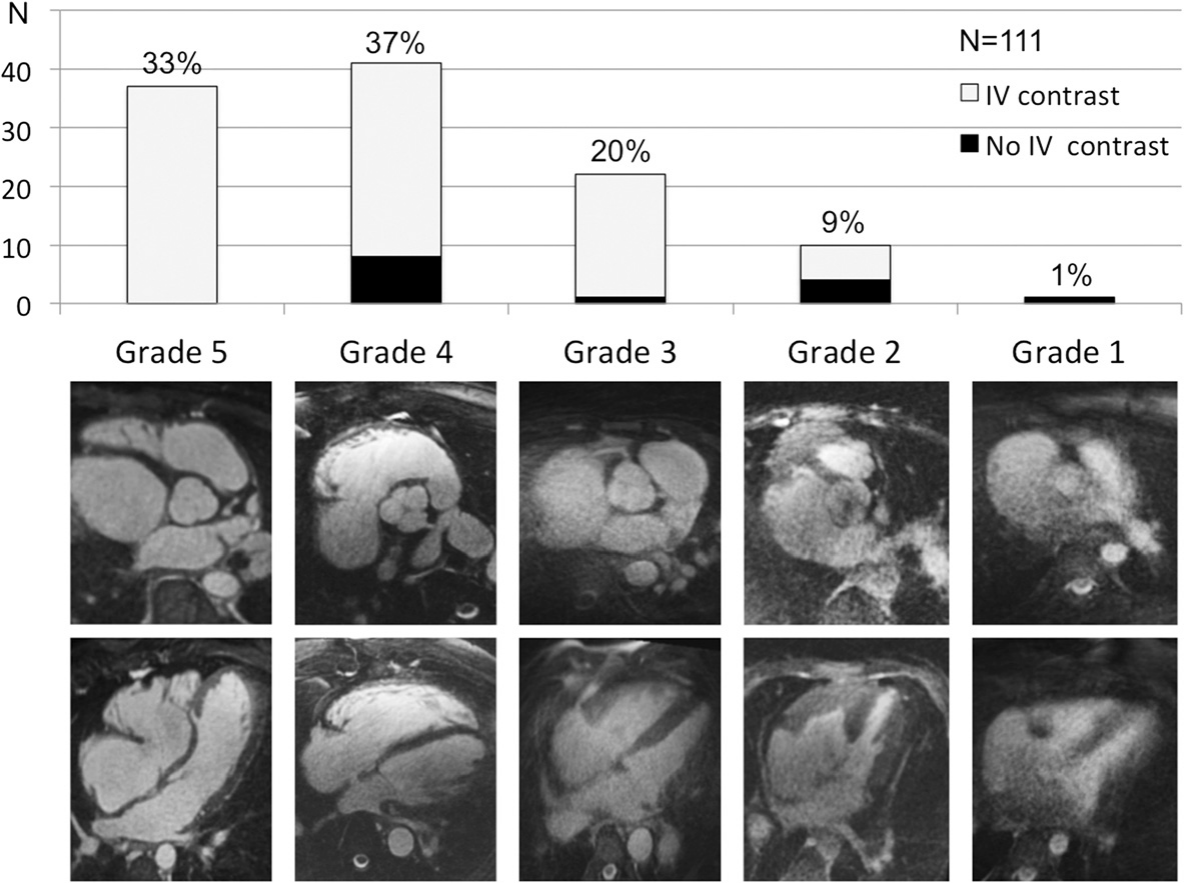
Image quality – Illustration of the 5-points scale for grading image quality (lower panel) and corresponding patient distribution (upper panel)

Image quality was graded independently by two experienced readers using a 5-points scale as previously described [15, 16]. Grade 5 corresponds to an excellent and grade 4 to a good diagnostic quality with mild blurring (Fig. 1), whereas grade 3 indicates diagnostic quality despite moderate blurring of cardiac and vascular structures, i.e. of the blood-myocardial interface of the left and right ventricle and the contours of the great vessels, respectively. Grade 2 indicates marked blurring of the structures, preventing a complete anatomical diagnosis. In grade 1, a dataset was considered non-diagnostic. Visualization of the coronary arteries was graded separately (see below).

### Coronary arteries

The left anterior descending, left circumflex (LCX) and right coronary artery (RCA) were considered “visible” if both their origin and their proximal segments were visually identified [17]. These criteria were considered sufficient to detect clinically relevant congenital abnormalities of the coronary arteries. All observers were blinded to the coronary anatomy if known by means of previous examinations.

### Great vessels

In 15 randomly chosen self-navigated 3D datasets, two experienced readers (P.M. and T.R.) measured the mean diameters (average of the maximal and minimal diameter) of the aorta and the pulmonary arteries at five and three different anatomical levels, respectively using Osirix v5.6 (Pixmeo, Bernex, Switzerland) software. The first investigator (P.M.) repeated the same measurements after one month.

### Factors influencing image quality

The influence on image quality was assessed for factors such as patient age, height, weight, HR, sinus arrhythmia (expressed as the SD of the RR-interval during acquisition), end-diastolic volume of the systemic ventricle, ejection fraction (EF, assessed by cine-CMR), type of malformation (complex vs non-complex), surgical correction of CHD, parameters of the 3D imaging sequence (see Table 1), and use of CM.

### Statistical analysis

Baseline characteristics were computed as mean ± SD and as percentages as appropriate. Comparisons according to quality grade were assessed with one-way ANOVA for continuous variables and Fisher exact test for categorical variables. Bivariate logistic regression was used to detect factors associated with poor image quality (quality grades 1–2 vs 4–5) and those factors with a significant (p < 0.10) bivariate association were re-tested in a stepwise multivariate logistic regression model.

For the assessment of inter-observer and intra-observer agreement for great artery diameter measurements, Bland-Altman analysis [18] was used and the coefficient of variation (CV%) was calculated as the SD expressed as the percentage of the mean. Statistical analysis was performed using STATA 13 (StataCorp, Texas, USA). A p-value <0.05 was considered statistically significant.

## Results

### Study population

Over the study period, 138 patients ≥2 years were referred for CMR at 1.5T with an established diagnosis of CHD. In 33 patients the self-navigated 3D acquisition was not performed due to time constraints resulting in a cohort of 105 patients. As six patients were scanned twice during the study period, 111 examinations were available for quality analysis. Baseline characteristics are summarized in Table 1. Mean age was 23.4 ± 12.2 years (range: 2.0–55.9 years), 27 % of patients were <16 years old and 55 % were male. Sedation was used in 10 children (age 4.3 ± 2.3 years). Cardiac malformation was complex in 44 % and 76 patients (68.5 %) had previous surgical correction, of those 44 (89.8 %) had complex malformations and 32 (51.6 %) had non-complex malformations.

### Segmental morphology and residual structural defects

With the self-navigated 3D acquisition alone, a correct segmental morphological description was achieved in 96.2 and 93.3 % by observer 1 and 2, respectively (Table 2, first-step analysis). Regarding the residual structural defects, 93.3 % (observer 2) to 95.2 % (observer 1) were correctly identified with the 3D self-navigated acquisition alone (Table 3, second-step analysis), and these numbers increased to 94.7 and 96.8 %, respectively, when only patients with diagnostic image quality (quality grades >2) were considered. Among the missed residual diagnoses listed in Table 3, only three (out of the total sample of 105 patients) were considered clinically relevant, including two septal defects (one ASD and one VSD) and one modified Blalock-Taussig shunt. The image quality in these three datasets was poor (quality grade 1 or 2).

**Table 3:** Rate of correct identification/exclusion of residual structural defects with 3D-self-navigated CMR

	N	Observer 1	Observer 2
Tetralogy of Fallot	19	19 (100 %)	18 (94.7 %)^a^
D-transposition of the great arteries	13	13 (100 %)	13 (100 %)
Fontan circulation	3	2 (66.7 %)^b^	2 (66.7 %)^b^
Syndromes associated with aortic dilatation	20	20 (100 %)	20 (100 %)
Coarctation of the aorta	7	6 (85.7 %)^c^	7 (100 %)
Ross operation	6	6 (100 %)	5 (83.3 %)^d^
Septum defects and abnormal venous returns	13	12 (92.3 %)^e^	11 (84.6 %)^f,^ ^g^
Other complex malformations	12	10 (83.3 %)^h,^ ^i^	10 (83.3 %)^j,^ ^i^
Other non-complex malformations	12	12 (100 %)	12 (100 %)
**All patients**	**105**	**100 (95.2 %)**	**97 (93.3 %)**

^a^Coronary artery abnormality suspected by CMR but not present

^b^Hypoplastic left heart syndrome with mitral atresia described intra-operatively by the surgeon (=reference diagnosis). On the CMR performed 8 years later, no mitral atresia was found. Cine sequences confirmed the presence of a hypoplastic but functional mitral valve

^c^Patent foramen ovale not recognized

^d^Coronary abnormality (left main stem re-implanted into the non-coronary sinus after Ross operation) not described by CMR

^e^Ostium secundum atrial septal defect not recognized

^f^Description of a RV outflow tract aneurysm that was not present

^g^Non-restrictive ventricular septal defect diagnosed by echocardiography, not recognized

^h^Modified Blalock-Taussig shunt not described on CMR

^i^Un-operated pulmonary valve described as atretic on echocardiography but considered as severely stenotic valve with hypoplastic pulmonary arteries on CMR

^j^Operated double outlet RV of Fallot type described as operated tetralogy of Fallot

### CMR image quality

**Fig. 2:**
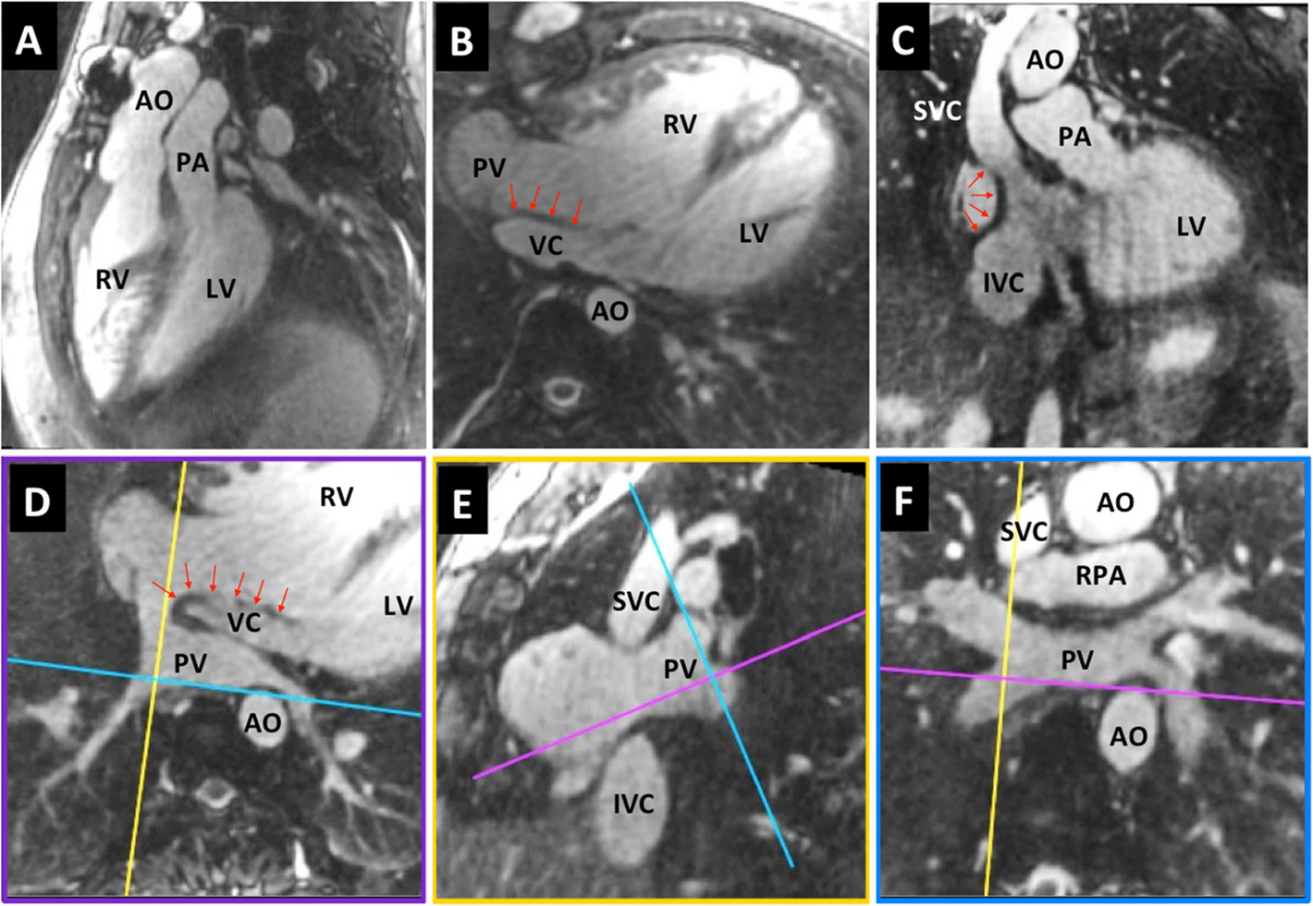
Offline reformats of the 3D data – Reformatted images from a 3D dataset acquired in a 25 years old patient with d-transposition of the great arteries and atrial switch palliation. Note the typical parallel course of the aorta and pulmonary artery (**a**) and the morphology of the systemic venous pathway (**b-c**). Complex morphology of the pulmonary venous pathway (**d-f**). Red arrows indicate inter-atrial baffle. AO-aorta; IVC-inferior vena cava; LV-left ventricle; PA-main pulmonary artery; PV-pulmonary veins; RA-right atrium; RPA-right pulmonary artery; RV-right ventricle; SVC-superior vena cava; VC-systemic venous conduit

The free-breathing self-navigated 3D acquisition was successful in all cases with a mean duration of 9.5 ± 3.3 min. Figure 2 shows an example of a self-navigated 3D CMR of a patient with d-transposition of the great arteries and atrial switch operation. Image quality was sufficient for a complete anatomical diagnosis (grades 3–5) in 90 % of examinations (Fig. 1) and 70 % had good to excellent quality (grades 4–5). Only 9 % had limited image quality allowing for a partial diagnosis and only one examination had completely non-diagnostic quality. Table 1 gives the distribution of the clinical and imaging characteristics for the 5 classes of image quality.

**Fig. 3:**
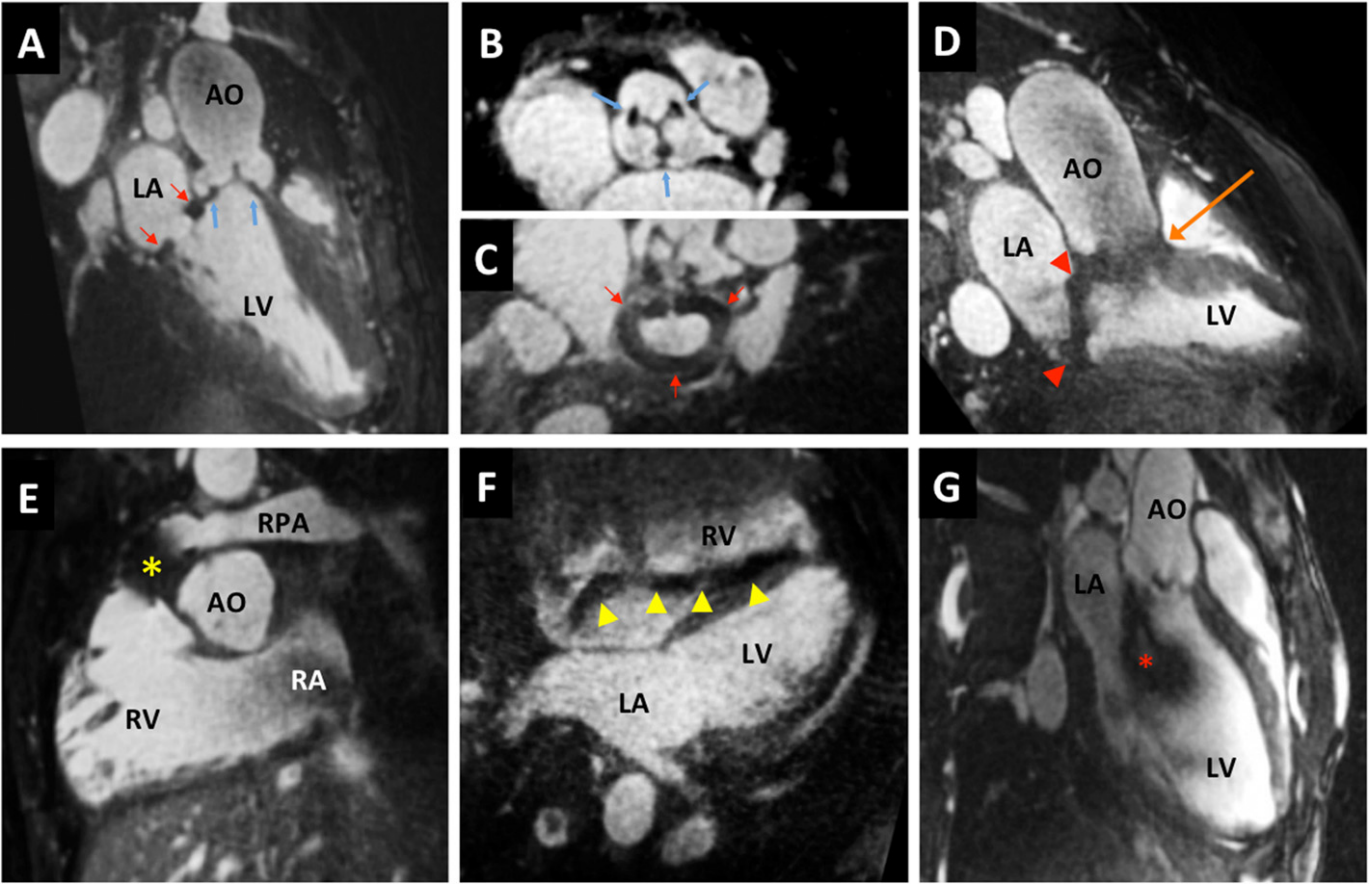
Examples of artifacts. **a**-**c** Bioprosthetic aortic valve (blue arrows) and mitral annuloplasty ring (red arrows) in a 30 years old patient; **d**: mechanical valves in aortic (orange arrow) and mitral (red arrowheads) position in a 33 years old patient; **e**: stent-valve in pulmonary position (*) in a 13 years old patient; **f**: pacemaker lead (yellow arrowheads) in a 50 years old patient; **g**: flow artifact related to severe aortic regurgitation (*). LA left atrium in a 19 years old patient. Other abbreviations as in Fig. 2

In the 97 examinations performed after CM injection the image quality was diagnostic in 94 %, of which 77 % were of good to excellent quality. While image quality improved by CM administration, focal artifacts were still identifiable, and were attributed to stents, prosthetic valves or pacemaker leads, or flow artifacts related to turbulent jets (Fig. 3).

### Factors that impact on image quality

To identify the potential factors that impact on image quality, their distribution in the two best groups (quality grades 4–5, n = 78) was compared versus the two worst groups (quality grades 1–2, n = 11). On bivariate logistic regression analysis, 9 factors were identified as associated with image quality (Table 4). On multivariate logistic regression analysis, only higher HR, younger age, lower EF, and lack of CM injection were associated with a decrease in image quality.

**Table 4:** Bivariate and multivariate logistic regression to identify factors associated with poor image quality (quality grades 1–2 vs quality grades 4–5)

	Bivariate analysis			Multivariate analysis		
	Odds ratio	*P*	95 % - confidence interval	Odds ratio	*P*	95 % - confidence interval
**Age (years)**	**0.98**	**ns**	**0.92–1.04**	**0.89**	**<0.05**	**0.8–0.99**
**Heart rate (bpm)**	**1.07**	**<0.01**	**1.02–1.12**	**1.11**	**<0.01**	**1.03–1.2**
Height (cm)	0.97	<0.05	0.95**–**0.99			
Weight (kg)	0.96	<0.05	0.93**–**0.99			
**EF (%)**	**5.8×10** ^**−6**^	**<0.01**	**1.3 × 10** ^**−9**^ **–0.03**	**1.2 × 10** ^**−10**^	**<0.01**	**2.4 × 10** ^**−17**^ **–6 × 10** ^**−4**^
Complex malformation	4.26	<0.05	1.05**–**17.35			
Surgical correction	0.31	0.07	0.08**–**1.11			
Acquisition window (ms)	0.96	<0.05	0.92–0–99			
Scan duration (s)	1.16	0.09	0.98**–**1.38			
**Use of IV contrast**	**0.14**	**<0.01**	**0.03–0.55**	**0.007**	**<0.01**	**0.0004–0.15**

**Fig. 4:**
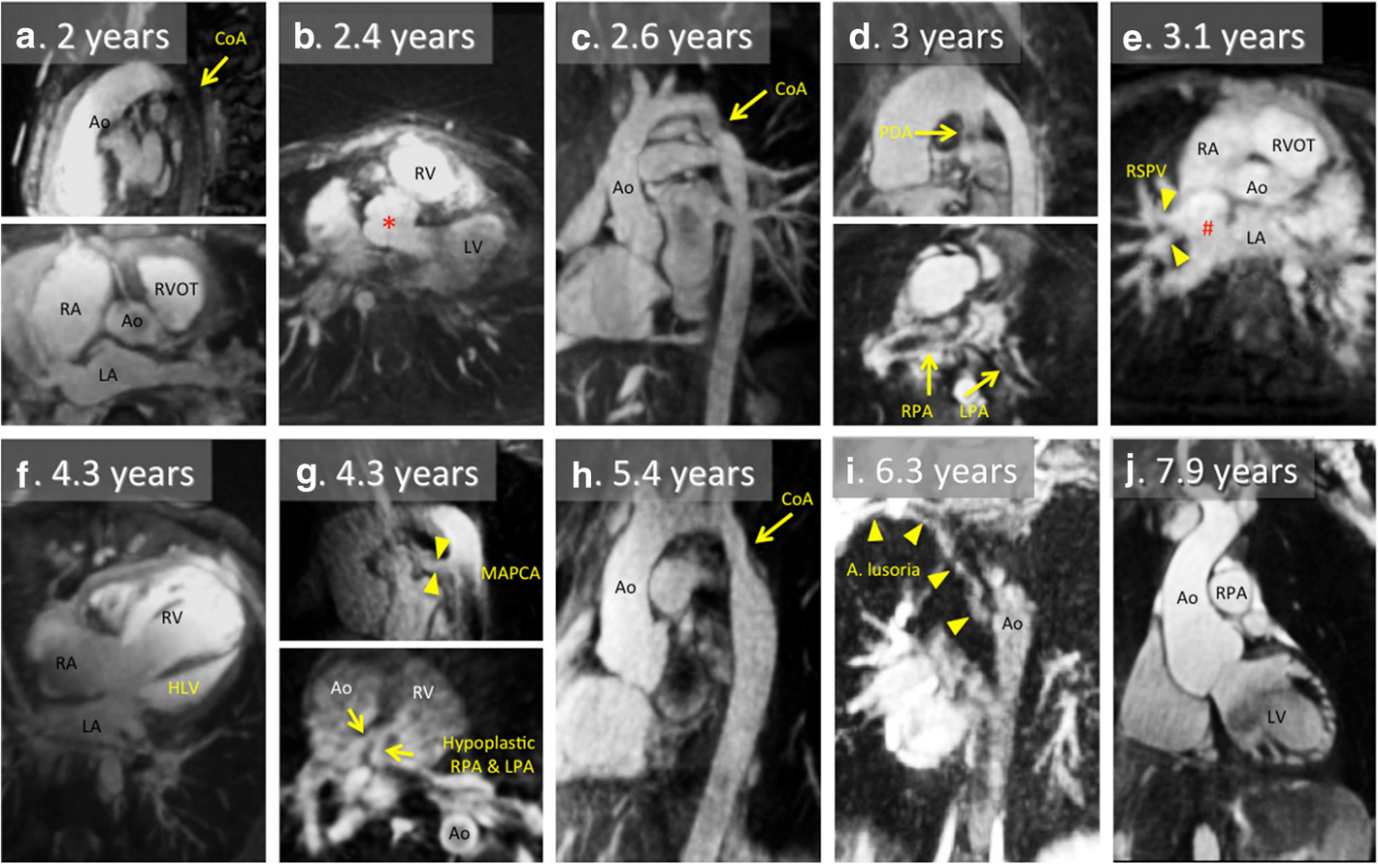
Representative 2D images reconstructed from the 3D self-navigated datasets in the 10 children presented on Table 6. **a**. Coarctation of the aorta. **b**. Operated type 1 truncus arteriosus; * indicates the aortic root (originally : common arterial trunk) overriding the interventricular septum. **c**. Coarctation of the aorta. **d**. Unoperated pulmonary atresia with open septum. E. Unoperated sinus venosus atrial septum defect; # indicates the defect – the right superior pulmonary vein drains into the proximal part of the superior vena cava. F. Hypoplastic left heart syndrome (after Norwood operation). G. Unoperated pulmonary atresia with open septum. H. Coarctation of the aorta. I. Aberrant right subclavian artery. J. Marfan syndrome with aortic root dilatation.CoA - aortic coarctation ; HLV - hypoplastic LV ; LA - left atrium ; LPA - left pulmonary artery ; PDA - patent ductus arteriosus ; MAPCA - major aorto-pulmonary collateral artery ; RSPV - right superior pulmonary vein ; RPA - right pulmonary artery ; RVOT - right ventricular outflow tract. Other abbreviations as in Fig. 2

To further characterize the performance of the self-navigated 3D sequence in the pediatric population, we compared the baseline characteristics and the obtained image quality according to quintiles of age (Table 5). The children (2 – 14 years, 1st quintile) had a significantly lower height and weight and a higher HR. The acquisition window was significantly shorter as it was individually adapted to the faster HR. The rate of diagnostic quality obtained with the self-navigated 3D sequence was not significantly different in children and in adults, although good to excellent quality datasets (quality grade 4 and 5) were more frequently obtained in adult patients. The rate of coronary artery detection was high in all age groups. To illustrate the performance of the sequence in the small children population, the baseline characteristics as well as representative images of the 10 youngest patients (2–8 years) are presented in Table 6 and Fig. 4.

**Table 5:** Baseline characteristics of 111 datasets according to age quintiles

Age	years *range*	8.4 ± 4.3	17.0 ± 1.8	21.4 ± 1.6	28.4 ± 3.4	43.0 ± 6.2	*p*
		*2.0–13.8*	*14.1–19.5*	*19.6–23.6*	*24.1–34.2*	*35.2*–*55.9*	
		N = 23	N = 22	N = 22	N = 23	N = 21	
Male gender	N (%)	10 (43 %)	15 (68 %)	9 (41 %)	13 (57 %)	14 (67 %)	0.23
HR	bpm	82.8 ± 16.6	78.4 ± 12.5	69.6 ± 9.5	67.9 ± 10.5	77.9 ± 16.0	**<0.001**
Height	cm	129.8 ± 32.4	166.1 ± 12.9	169.5 ± 10.7	172.4 ± 9.2	166.4 ± 10.4	**<0.001**
Weight	kg	29.8 ± 16.2	55.5 ± 12.1	66.0 ± 16.5	71.0 ± 13.1	68.8 ± 14.9	**<0.00** **1**
Complex malformation	N (%)	10 (43 %)	9 (41 %)	9 (41 %)	11 (48 %)	10 (48 %)	0.98
Corrected malformation	N (%)	13 (57 %)	15 (68 %)	18 (82 %)	18 (78 %)	12 (57 %)	0.24
Acquisition window	ms	57.9 ± 28.0	69.8 ± 21.0	71.2 ± 15.2	76.3 ± 18.0	66.0 ± 18.1	**<0.05**
Scan duration	min	10.9 ± 3.5	9.1 ± 4.4	9.2 ± 1.9	9.0 ± 2.5	9.0 ± 2.2	0.19
Use of contrast	N (%)	18 (78 %)	21 (95 %)	20 (91 %)	22 (96 %)	16 (76 %)	0.15
Diagnostic quality	N (%)	19 (83 %)	21 (95 %)	21 (95 %)	22 (96 %)	17 (81 %)	0.28
Good/excellent quality	N (%)	11 (48 %)	18 (82 %)	20 (91 %)	17 (74 %)	12 (57 %)	**<0.01**
LAD visualized	N (%)	20 (87 %)	20 (91 %)	22 (100 %)	23 (100 %)	18 (86 %)	0.12
LCx visualized	N (%)	18 (78 %)	19 (86 %)	21 (95 %)	21 (91 %)	18 (86 %)	0.49
RCA visualized	N (%)	23 (100 %)	22 (100 %)	21 (95 %)	23 (100 %)	20 (95 %)	0.34

**Table 6:** Characteristics of pediatric patients aged <8 years

Patient	age *(years)*	Diagnosis	Surgical correction	Heart rate *(bpm)*	Weight *(kg)*	Temporal resolution *(ms)*	Scan duration *(min)*	Contrast injected	N coronary visualized	Quality grade
1	2	Aortic coarctation + VSD	yes	91	12	30	16.5	yes	3	4
2	2.4	Truncus arteriosus type 1	yes	116	10	18	16.4	yes	3	3
3	2.6	Aortic coarctation	yes	87	14	48	10.3	yes	3	5
4	3	Pulmonary atresia with open septum	no	110	9	21	18.5	yes	3	2
5	3.1	Sinus venosus ASD + abn venous return	no	93	13	30	12.8	yes	3	3
6	4.3	Hypoplastic left heart syndrome	yes	68	14	33	14.7	yes	3	3
7	4.3	Pulmonary atresia with open septum	no	111	12	30	12.5	yes	3	2
8	5.4	Aortic coarctation	yes	108	17	30	13.9	yes	3	4
9	6.3	Aberrant right subclavian artery (A lusoria)	no	87	16	21	11.8	yes	3	3
10	7.9	Marfan syndrome	no	59	26	96	6.3	no	3	5

In the population that did not undergo the self-navigated CMR acquisition (n = 33), HR was slightly lower than in the study population (68.4 ± 12.3 vs 75.4 ± 14.3bpm, p < 0.05), while no differences were found for the other factors that impact on image quality, i.e. age, EF, and use of CM.

### Coronary arteries

**Fig. 5:**
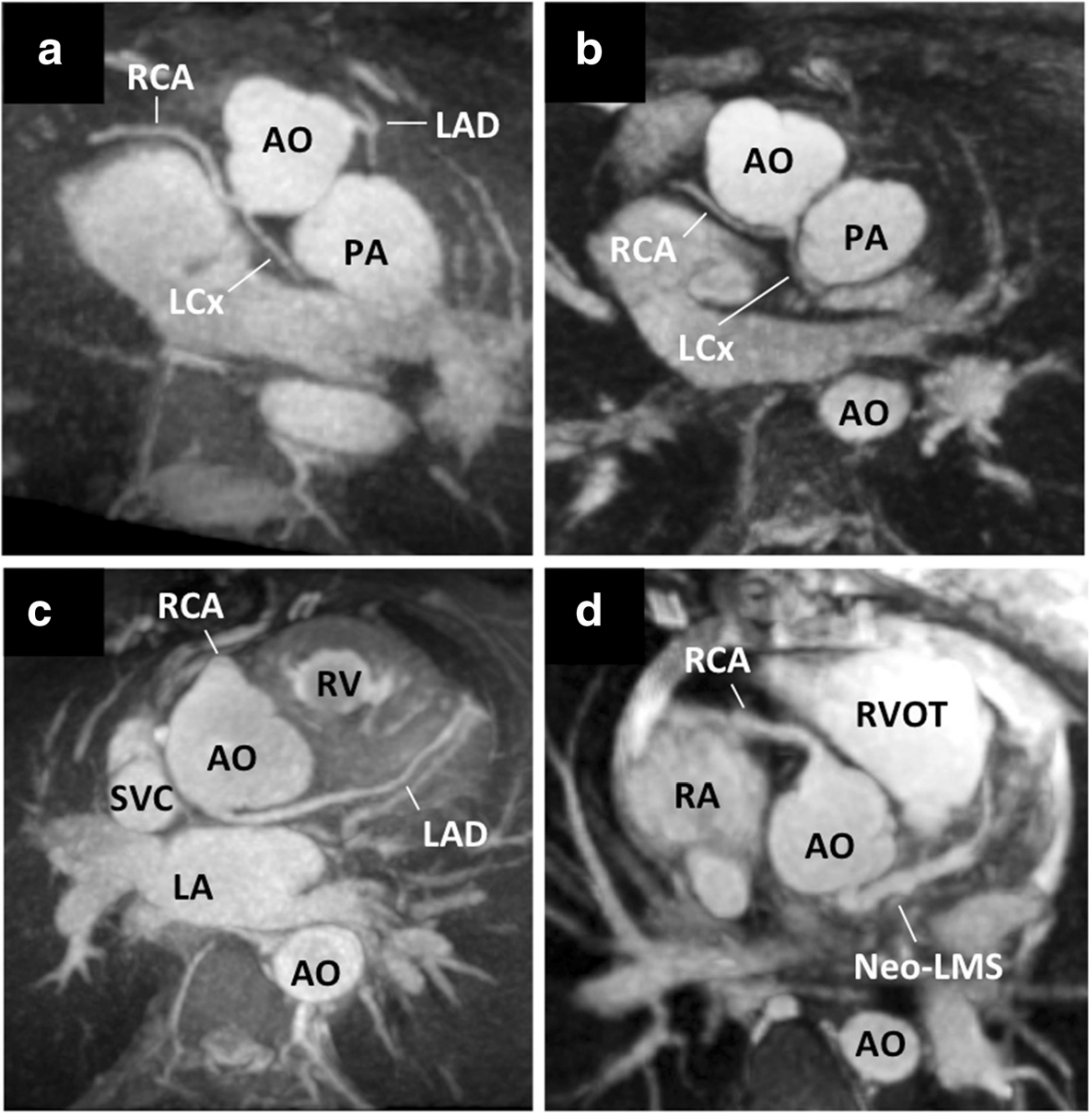
Coronary artery anomalies – Four cases of abnormal coronary artery course and origins were detected (3D reconstructions performed using Osirix v5.6, Pixmeo, Bernex, Switzerland): LCX originating from the left-hand facing sinus together with the RCA in two patients (both 19 years old) with d-transposition of the great arteries (Panels **a** and **b**), left coronary artery originating from the non-coronary sinus in a 30 years old patient (**Panel c**), surgically reconstructed left main stem for abnormal left coronary artery originating from the pulmonary artery (ALCAPA) syndrome in a 20 years old patient (**Panel d**). LAD-left anterior descending artery; LCX-left circumflex artery; LM-left main stem; RCA-right coronary artery; other abbreviations as in Fig. 2

The origin and the proximal course were visualized in 93 %, 87 %, and 98 % of the LAD, LCX, and RCA, respectively (Table 7). The RCA was detected even in images of lower overall quality (grade < 3). In the four patients (3.6 %) with a known coronary abnormality, these abnormalities were clearly visualized (Fig. 5).

### Great vessels

**Fig. 6:**
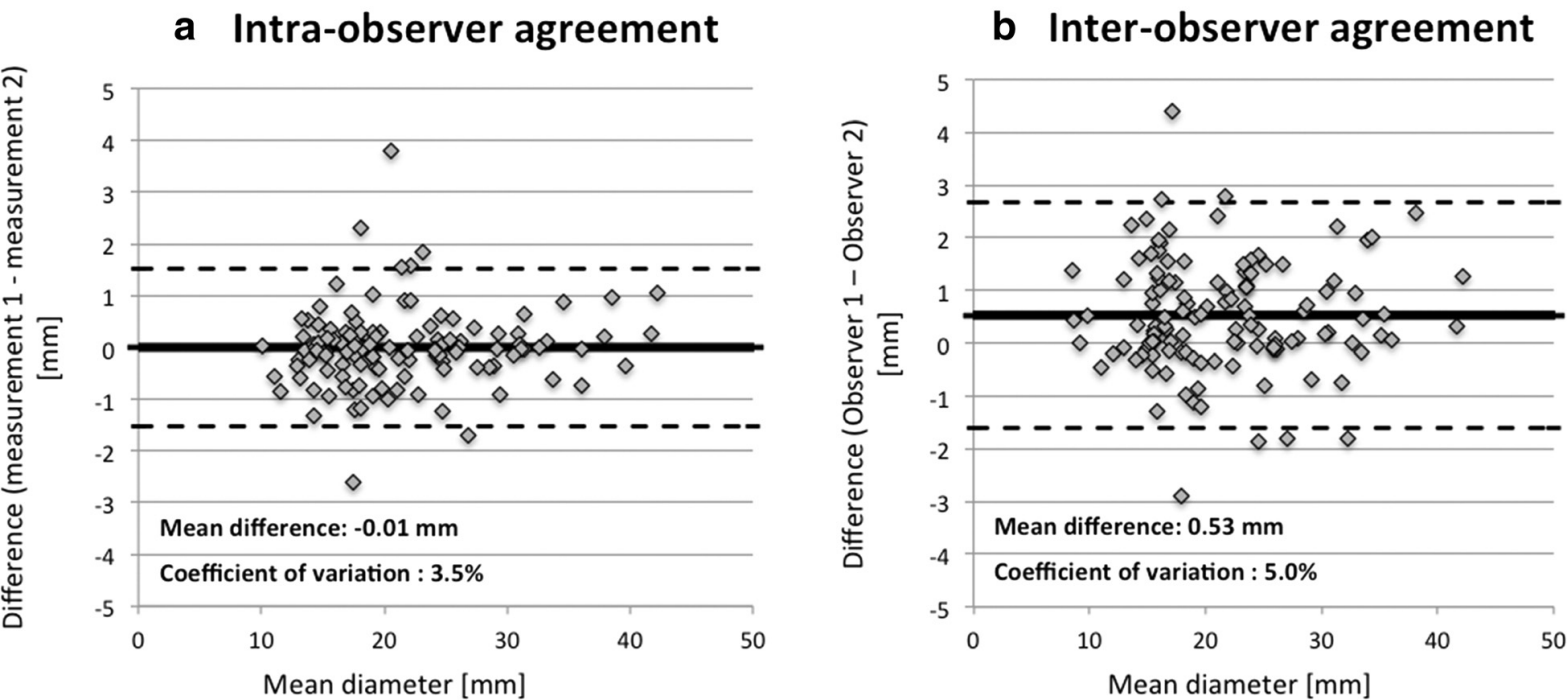
Reproducibility – Bland-Altman analysis for the intra-observer (Panel **a**) and inter-observer (Panel **b**) agreement for great vessels measurements. Measurements of the aorta were performed at 5 different levels (sinus of Valsalva, ascending aorta, proximal aortic arch, distal aortic arch, descending aorta at the level of the diaphragm) and measurement of the pulmonary arteries at 3 different levels (main pulmonary artery, right and left pulmonary arteries)

Using 3D self-navigated datasets, the reproducibility of great artery diameter measurements at eight different sites was high, with an excellent agreement both for intra-observer (bias −0.01 mm; CV% 3.5 %) and inter-observer analysis (bias 0.53 mm; CV% 5.0 %), as shown on Fig. 6.

## Discussion

### Accurate assessment of segmental morphology and identification of residual uncorrected structural defects

We report the first clinical experience with a self-navigated 3D CMR imaging technique in an unselected population of CHD patients, which allows performing a correct description of the segmental cardiac anatomy in 93 to 96 % of patients. This performance is considered excellent, as the 3D datasets were used exclusively, and we can expect that the combination of the self-navigated 3D acquisition with conventional functional imaging will increase the diagnostic accuracy of CMR in this population. Only three missed residual diagnoses out of the total sample of 105 patients were considered clinically relevant, including two septal defects (one ASD and one VSD) and one modified Blalock-Taussig shunt. Despite an inadequate quality (grade 1 or 2) in all these three patients, the sensitivity of the current version of the self-navigated whole-heart 3D CMR appears insufficient to confidently assess the thin inter-atrial septum or to detect small VSD’s and we would not recommend this technique as the only imaging approach to diagnose or exclude such malformations.

### Image quality and robustness of the self-navigated high-resolution 3D CMR acquisition

The self-navigation sequence, when combined with CM administration is characterized by a high success rate yielding diagnostic quality in over 94 % of examinations. Quantitative measure of image quality included the high inter-observer and intra-observer agreement for great vessels diameter measurements (Fig. 6). The quality of the self-navigated 3D sequence was further demonstrated by the reliable delineation of the origin and proximal course of the coronary arteries, allowing for an efficient detection of aberrant coronary arteries (Fig. 5).

### Factors that influence image quality

In 11 examinations image quality was insufficient for a complete assessment of all cardiac and vascular structures. We identified 4 factors (young age, high HR, low EF and lack of CM injection) that were independently associated with reduced image quality.

We hypothesize that the association between young age and poor image quality might be explained by a lower tracking efficiency of the self-navigation technique for the smaller cardiac silhouette as the motion detection algorithm is based on the identification of the ventricular blood pool signal. In addition, artifacts related to rapid and turbulent flow are sometimes observed with the 3D self-navigated sequence (Fig. 3g); while these artifacts are rarely large enough to blur the contours of large adult vascular structures, they might more often obscure small-sized vessels in children and result in overall reduction of image quality. Furthermore, a high HR, which typically occurs at younger age reduces the duration of the motionless mid-diastolic period and may favor image artifacts related to unsuppressed residual cardiac motion despite an individual adaptation of the acquisition window to the HR. Even if a good to excellent image quality was more frequently obtained in adult patients, the rate of diagnostic image quality obtained with the sequence was not significantly different in children and in adults. For this reason, we believe it is justified to recommend this technique also for examinations of children.

The association of low EF with poor image quality was somewhat surprising as dysfunction is expected to cause less cardiac motion during the cardiac cycle. However, systolic dysfunction occurs in association with diastolic dysfunction and as myocardial relaxation is prolonged, the mid-diastolic period shortens or disappears, potentially limiting the efficiency of cardiac gating. It is also known that irregular breathing patterns may be more prevalent among heart failure patients with lower EF [19, 20] and therefore it might be speculated that this association between a lower EF and more irregular breathing patterns might also be related to the reduced image quality observed in our CHD population. With the current version of the self-navigation pulse sequence, respiratory motion correction is performed in the superior-inferior direction only, which is the main direction of displacement of the heart during the respiratory cycle. With larger diaphragmatic excursions, the degree of anterior-posterior, lateral, and rotational displacements of the heart are likely to increase [21, 22], but are not yet corrected for, potentially causing additional blurring.

Finally, we observed that the use of CM was associated with improved image quality. This might be simply explained be a higher signal-to-noise ratio, but the enhancement of the blood pool signal within the ventricles could also improve the tracking of the cardiac silhouette, resulting in a better correction of respiratory motion.

Image quality appeared different in patients with complex vs non-complex malformations as shown on Table 1, with 16.3 % of insufficient quality scans in complex malformations vs 4.8 % in non-complex malformations. Patients with complex malformations had an age similar to that of patients with non-complex malformations (23.1 ± 1.7 vs 23.5 ± 1.6 years, p = 0.85) but a lower EF of the systemic ventricle (EF 54.7 ± 1.4 % vs 61.0 ± 0.8 %, p < 0.001). Accordingly we observed a univariate correlation between complex malformation and image quality, which disappeared after adjustment for EF.

### Potential strategies to control quality-associated imaging factors

Several strategies could be considered to further improve the image quality. In patients with a high HR, a shorter acquisition window can be used to better “freeze” the cardiac motion, or pharmacological HR reduction can be achieved. Motion correction algorithms should be further developed to correct for the position of the heart in all three spatial directions rather than in the superior-inferior direction only as performed in this prototype pulse sequence. New self-navigation-based approaches have recently been reported allowing for 2D [23] or even for a 3D respiratory motion correction [24, 25]. The present study underlines the ease of use of self-navigation application, as no extra-navigators have to be placed on the liver dome, and more advanced 2D or 3D self-navigation techniques are likely to further improve the quality of the presented approach. Selective removal of k-space profiles that originate from respiratory outlier positions of the diaphragm can also be performed as an alternative strategy. Finally, CM injection [26] should be strongly considered in patients where a lower image quality can be expected due to young age, high HR, or low EF.

### Comparison with other 3D CMR approaches

It was not the aim of this study to directly compare self-navigation to conventional diaphragmatic navigation for the acquisition of 3D datasets of the heart. Nevertheless, the presented results suggest that self-navigated 3D CMR is a robust technique when used in a large number of CHD patients. In a previous report on the use of navigator-gated 3D whole heart acquisition in CHD patients, Sørensen et al. [16] reported that 21 out of 52 datasets (40 %) were excluded because of an inadequate image quality. In a pediatric population, Hussain et al. [27] described the performance of a dual-phase, i.e. systolic and diastolic navigator-gated 3D acquisition for the assessment of the great vessels including the caval veins and the pulmonary veins. Image quality was sufficient to measure the diameter of the great vessels in all segments in 31/50 (62 %) patients when acquired in systole and in 26/50 (52 %) patients when acquired in mid-diastole. The young age of the study subjects may explain the lower success rate in that study, which is in line with the current findings that younger age correlates with reduced image quality. Although the caval and pulmonary veins were not included in the present study, the presented self-navigated 3D CMR compares favorably with these historical data using 1D diaphragmatic navigators, as the great vessels could be analyzed in all patients with good accuracy.

### Limitations

Small volunteer cohorts [10, 12] already showed that self-navigation resulted in an equivalent or superior image quality as compared with conventional navigation for 3D whole-heart acquisitions. A head to head comparison of the two techniques was not performed in this selected cohort of CHD patients. Such a comparison would be of high interest for future investigations.

The current study did not include children below two years of age. The performance of the 3D self-navigated sequence is therefore not known for newborns and very small children, and further studies are needed in this specific age category.

## Conclusions

In patients with CHD, the CM-enhanced self-navigated 3D CMR yielded a diagnostic image quality in 94 % of patients, 77 % of which were of good to excellent image quality. This was reflected by a high success rate in describing segmental cardiac anatomy and in detecting residual cardiac malformations. Considering its high spatial resolution, and its high image quality and robustness in a large patient population, self-navigated 3D CMR represents a valuable expansion of the current imaging armamentarium for the cardiac assessment of CHD in adults and children.
