# Erratum to: Single centre experience of the application of self navigated 3D whole heart cardiovascular magnetic resonance for the assessment of cardiac anatomy in congenital heart disease

**DOI:** 10.1186/s12968-015-0178-1

**Published:** 2015-10-06

**Authors:** Pierre Monney, Davide Piccini, Tobias Rutz, Gabriella Vincenti, Simone Coppo, Simon C. Koestner, Nicole Sekarski, Stefano Di Bernardo, Judith Bouchardy, Matthias Stuber, Juerg Schwitter

**Affiliations:** Division of Cardiology and Cardiac MR Center, University Hospital of Lausanne (CHUV), Lausanne, Switzerland; Advanced Clinical Imaging Technology, Siemens Healthcare, Lausanne, Switzerland; Department of Radiology, University Hospital and University of Lausanne, Lausanne, Switzerland; Center for Biomedical Imaging and Center for Cardiovascular Magnetic Resonance Research, University of Lausanne, Lausanne, Switzerland; Pediatric Cardiology Unit, University Hospital of Lausanne (CHUV), Lausanne, Switzerland

Following publication of the original version of this article [1], it was found that Table seven was missing due to a Production error. The table is now provided below as Table 1.

**Table 1:** Detection of the origin and proximal course of the coronary arteries

Coronary artery		Total cohort	Quality grade 1	Quality grade 2	Quality grade 3	Quality grade 4	Quality grade 5	*P*
		*N* = 111	*N* = 1	*N* = 10	*N* = 22	*N* = 41	*N* = 37	
LAD	*N* (%)	103 (93 %)	0 (0 %)	6 (60 %)	20 (91 %)	40 (98 %)	37 (100 %)	<0.001
LCX	*N* (%)	97 (87 %)	0 (0 %)	6 (60 %)	18 (82 %)	37 (90 %)	36 (97 %)	<0.01
RCA	*N* (%)	109 (98 %)	1 (100 %)	10 (100 %)	21( 95 %)	40 (98 %)	37 (100 %)	0.75
